# Regulation of Krüppel-Like Factor 4 (KLF4) expression through the transcription factor Yin-Yang 1 (YY1) in non-Hodgkin B-cell lymphoma

**DOI:** 10.18632/oncotarget.26745

**Published:** 2019-03-15

**Authors:** Mario Morales-Martinez, Alberto Valencia-Hipolito, Gabriel G. Vega, Natividad Neri, Maria J. Nambo, Isabel Alvarado, Ivonne Cuadra, Marco A. Duran-Padilla, Otoniel Martinez-Maza, Sara Huerta-Yepez, Mario I. Vega

**Affiliations:** ^1^ Molecular Signal Pathway in Cancer Laboratory, UIMEO, Oncology Hospital, Siglo XXI National Medical Center, IMSS, México City, México; ^2^ Unidad de Posgrado, Facultad de Medicina Universidad Nacional Autónoma de México, México City, México; ^3^ Unidad de Investigación en Enfermedades Oncológicas, Hospital Infantil de México “Federico Gómez” S.S.A, México City, México; ^4^ Department of Hematology, Oncology Hospital, National Medical Center, IMSS, México City, México; ^5^ Servicio de Anatomía Patológica, Hospital de Oncología, Centro Médico Nacional Siglo XXI, IMSS, México City, México; ^6^ Servicio de Patología, Hospital General de México “Eduardo Liceaga”, Facultad de Medicina de la UNAM, México City, México; ^7^ Department of Obstetrics and Gynecology, Jonsson Comprehensive Cancer Center, UCLA AIDS Institute, David Geffen School of Medicine, University of California, Los Angeles, California, USA; ^8^ Department of Medicine, Hematology-Oncology Division, Greater Los Angeles VA Healthcare Center, UCLA Medical Center, Jonsson Comprehensive Cancer Center, Los Angeles, California, USA; ^9^ Department of Microbiology, Immunology, and Molecular Genetics, Jonsson Comprehensive Cancer Center, UCLA AIDS Institute, David Geffen School of Medicine, University of California, Los Angeles, California, USA

**Keywords:** KLF4, hematological malignancies, non-Hodgkin lymphoma, YY1, transcriptional regulation

## Abstract

Krüppel-Like Factor 4 (KLF4) is a member of the KLF transcription factor family, and evidence suggests that KLF4 is either an oncogene or a tumor suppressor. The regulatory mechanism underlying KLF4 expression in cancer, and specifically in lymphoma, is still not understood. Bioinformatics analysis revealed two YY1 putative binding sites in the KLF4 promoter region (-950 bp and -105 bp). Here, the potential regulation of KLF4 by YY1 in NHL was analyzed. Mutation of the putative YY1 binding sites in a previously reported system containing the KLF4 promoter region and CHIP analysis confirmed that these binding sites are important for KLF4 regulation. B-NHL cell lines showed that both KLF4 and YY1 are co-expressed, and transfection with siRNA-YY1 resulted in significant inhibition of KLF4. The clinical implications of YY1 in the transcriptional regulation of KLF4 were investigated by IHC in a TMA with 43 samples of subtypes DLBCL and FL, and all tumor tissues expressing YY1 demonstrated a correlation with KLF4 expression, which was consistent with bioinformatics analyses in several databases. Our findings demonstrated that KLF4 can be transcriptionally regulated by YY1 in B-NHL, and a correlation between YY1 expression and KLF4 was found in clinical samples. Hence, both YY1 and KLF4 may be possible therapeutic biomarkers of NHL.

## INTRODUCTION

Lymphoma is the sixth most common cause of cancer in terms of global incidence. There are two major types of lymphoma: Hodgkin lymphoma (HL) (40%) and non-Hodgkin lymphoma (NHL) (60%). Risk factors associated with lymphomas include physical, biological, and immunodeficiency factors as well as chemical agents [1].

Immunophenotypic analysis is very important for the sub-classification of lymphomas according to the recent classification of hematological malignancies by the World Health Organization (WHO) [2]. It is possible to correctly sub-classify B-cell lymphomas in most cases using specific biomarkers, but in some cases, it is hard to differentiate these biomarkers between normal cells and malignant cells undergoing proliferation [3] The identification of transcription factors involved in the development of a lymphoid progenitor is of interest, with important therapeutic implications for a great variety of conditions such as leukemia and lymphoma [3]. Oncogenes implicated in lymphoma pathogenesis include c-myc, [4, 5] Bcl-1 [6], Bcl-2 [4], Bcl-3 [7], and Bcl-6 [6, 8].

The transcription Krüppel like factor 4 (KLF4) can activate or suppress the transcription of various genes [9]. Its alteration leads to unregulated proliferation and differentiation in gastric epithelium and cell in the colon [10]. In breast cancer KLF4 has been reported to have an important role in oncogenesis, as well as in the maintenance of trunk type characteristics, it has also been reported as a promoter of cell invasion migration [11] While in pre-B cells, an important participation in the regulation of the cell cycle has been established, overexpression of KLF4 can induce arrest of the cell cycle and apoptosis [12].

Clinical evidence suggests that KLF4 is a potent tumor repressor, but in addition, KLF4 recently has been seen to act as an oncogenic element in various cancers [13]. KLF4 expression can be epigenetically regulated by hyper-methylation of its promoter in gastrointestinal cancer. Recently, it was reported that Cdx2 is a transcriptional regulator of the KLF4 promoter [14], and its autoregulation is mediated by miRNA-206 [15]. In addition, miR-10b [16], miRNA-346 [17], and miRNA-7 regulate KLF4 expression via an autoregulatory feedback loop [18].

Recent studies have shown that KLF5 binds to consensus elements like KLF4, including one that is present in the KLF4 promoter; however, it has been reported that KLF4 is an activator element, while KLF5 represses the activity of the KLF4 promoter. Additionally, KLF4 was reported to be an inhibitory transcriptional factor of KLF11 [16]. The factor FOXO was identified as a regulator of KLF4 transcription, suppressing B-cell proliferation [19]. Nevertheless, to date the regulatory mechanism underlying KLF4 expression in hematological malignancies such as NHL remains unknown [13].

Recently, we have shown that KLF4 is expressed in pediatric lymphomas, and this expression is higher in the subtype Burkitt and correlates with a poor prognosis and low patient survival [20]. In addition, the transcription factor YY1 is expressed in lymphomas, and computational analysis has shown that its expression correlates with poor survival in lymphoma patients [21]. Nevertheless, the role of these transcription factors in the pathogenesis of lymphoma is not clear and given their possible co-expression and correlation with poor prognosis, it is plausible to think that there is a relationship between these two transcription factors. Our first approach hypothesized that there is possible transcriptional regulation between these two transcription factors, and we presumed that YY1 can regulate the expression of KLF4. Understanding the regulatory mechanism underlying KLF4 expression and its implications in lymphomagenesis, as well as its relationship with the transcription factor YY1, is important for diagnostic and prognostic purposes.

This hypothesis was tested by various means. (1) A bioinformatics analysis of the KLF4 promoter region was performed. 2) *In silico* analyses were corroborated by using the CHIP (chromatin immunoprecipitation) experimental technique to demonstrate the functionality of putative binding sites for YY1 in the KLF4 promoter. 3) A reporter system was used to investigate the transcriptional regulation of YY1 and evaluate potential binding sites of YY1 by site-directed mutagenesis. 4) The biological role of KLF4 regulation by YY1 was analyzed via the use of siRNA, and KLF4 expression was determined. 5) The clinical implications of YY1 in the transcriptional regulation of KLF4 were correlated via IHC in a tissue microarray with B-NHL samples and by western blotting in B-NHL cell lines. 6) The data obtained with tumor tissues were validated by performing bioinformatics analysis.

## RESULTS

### Transcriptional regulation of the KLF4 protein by YY1 in lymphoma cell lines

**Figure 1:**
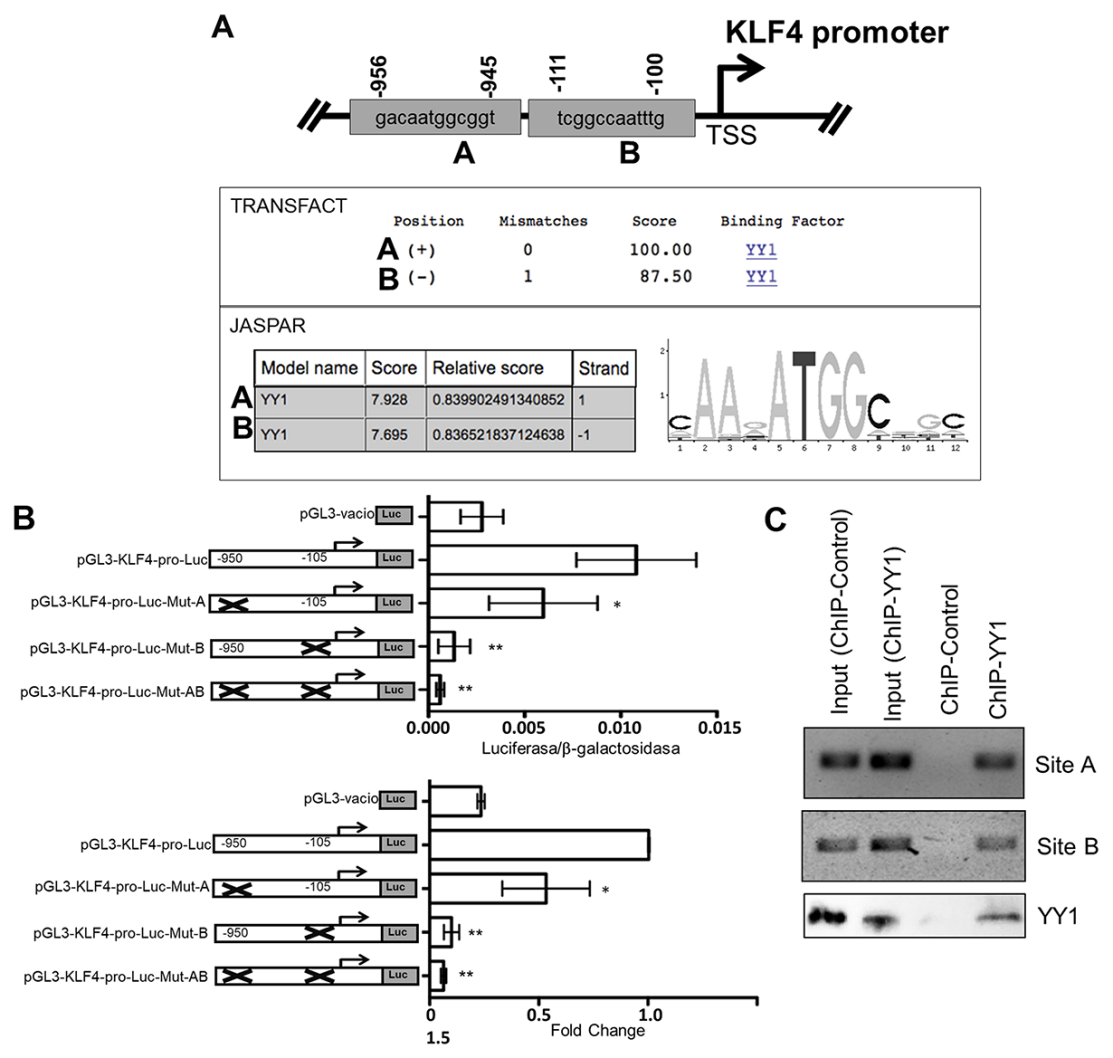
Bioinformatics analysis of the sequence of the promoter region of the KLF4 gene (**A**) Two potential binding sites for the transcription factor KLF4 obtained after bioinformatics analysis using two online servers, JASPAR and TRANSFACT, are displayed. The region from −2000 to +160 bp in the KLF4 gene was analyzed for Site Transcription Initiation (SIT). A weight matrix obtained from the JASPAR database for the transcription factor KLF4 is displayed. (**B**) Putative binding YY1 sites in the KLF4 promoter that are involved in regulating expression. Transfection assays were performed using the PC3 cell line to assess the effects of directed mutagenesis at each of the YY1 binding sequences, located at sites -950 bp and -105 bp in the promoter region of the KLF4 gene. The schematic shows each of the mutated sites, and the graph indicates normalized luciferase reporter gene expression levels obtained by measuring β-galactosidase via co-transfection with a reporter gene plasmid (top panel); fold changes are reported (bottom panel). The results are representative of three independent experiments (one-way ANOVA, ^*^p < 0.005, ^**^p < 0.001). (**C**) ChIP was conducted for each potential YY1 binding site in the KLF4 promoter. The results show that YY1 binds the promoter region of KLF4.

Based on independent findings regarding the expression of KLF4 and YY1 in lymphomas, we proposed that there is a correlation between these proteins. To probe the hypothesis, that there is transcriptional regulation between these proteins, we performed bioinformatics analyses to predict YY1 binding sites in the KLF4 promotor with the program TESS (Transcriptional Element Search System), which combines the TRANSFAC v6.0, JASPAR 20060301, IMD v1.1 and CBI/GibbsMat v1 databases. We analyzed 2000 nucleotides upstream (−2000 bp) of the start codon ATG to +160 nucleotides downstream (+160 bp) of the reported promoter region. We identified two possible binding sites, located at -950 bp and -105 bp with respect to the start codon for KLF4 gene transcription. (Figure 1A).

To determine if YY1 can regulate the expression of KLF4 through activation of its promoter, we evaluated the role of each binding site in regulating of the promoter region of the gene encoding KLF4. The KLF4 promoter region was cloned into the reporter plasmid pGL3 as described in the Materials and Methods. The reporter plasmid pGL3-KLF4-pro-luc was generated. Single or double mutation of the sites in the KLF4 promoter was performed. The mutants were designated pGL3-KLF4-MutA-pro-luc (site -950) and pGL3-KLF4-MutB-pro-luc (site -105) for the single mutants and pGL3-KLF4-MutAB-pro-luc for the double mutant. Reporter plasmids containing their respective mutations were transfected into the PC3 cell line, as a transfection model, as previously reported [22]. Transfection was performed using liposomes as described in the Materials and Methods. Figure 1B shows the luciferase results. For pGL3-KLF4-pro-luc, which contains the complete promotor of KLF4, the luciferase/B-galactosidase results were significant at ^*^p < 0.05, while the results with the plasmid pGL3-KLF4-MutA-pro-luc were significant at ^*^p < 0.01. However, the most dramatic effect observed with the reporter gene (luciferase) was obtained with the plasmid pGL3-KLF4-MutB-pro-luc, for which luciferase/B-galactosidase activity was almost zero, with similar results to those observed with the empty plasmid, and with pGL3-KLF4-MutA-pro-luc, which exhibited approximately half of the luciferase/B-galactosidase activity produced by the plasmid pGL3-KLF4-MutB-pro-luc (^*^p < 0.005). This result was corroborated by the luciferase/B-galactosidase activity observed with the plasmid containing double mutants (Figure 1B top plot). When site A and B were mutated, the activity of the reporter plasmid was affected, and the fold change with respect to the control is shown (Figure 1B bottom plot). These results show that the sites at −950 bp and −105 bp play important roles in the positive regulation of KLF4 by YY1.

To confirm the interaction of the transcription factor YY1 and the promoter region of KLF4, ChIP assays were performed. Chromatin from the Ramos cell line was used. For immunoprecipitation, an anti-YY1 antibody was used, and then segments were amplified by PCR using specific oligonucleotides for each possible binding site of YY1 in the KLF4 promoter region. The results are shown in Figure 1C, and we observed that YY1 binds directly to two sites in the KLF4 promoter. Non-immunoprecipitated chromatin was used as a positive control, and control IgG was used as a negative control. ChIP-WB is shown as efficiency of the IP YY1 and control (Figure 1C bottom). This result shows for first time that YY1 can positively regulate KLF4 expression.

### Inhibition of YY1 expression impacts KLF4 expression

**Figure 2:**
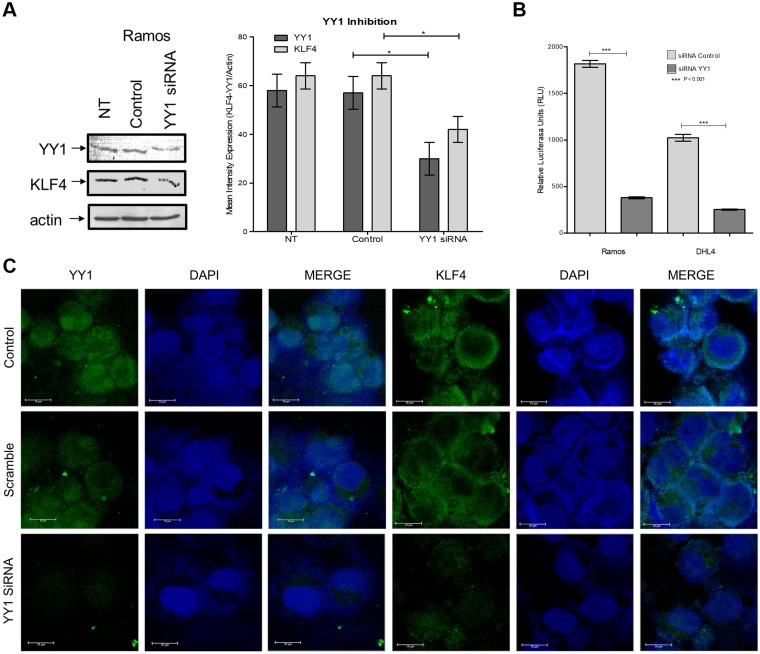
Analysis of expression inhibition by YY1 using siRNA (**A**) Ramos cells were transfected with YY1 siRNA or a control siRNA, and the expression of YY1 and KLF4 was evaluated by western blot (right panel) and densitometry analysis (left panel) (*p* = 0.0462). (**B**) Cells transfected with KLF4-Luc promoter (Switchgear), were treated with siRNA YY1 and Luc activity determinate. (**C**) Cells transfected with siRNA YY1 were analyzed by Immunofluorescence, and YY1 and KLF4 expression were determined. The results show clear inhibition of the total and nuclear expression of KLF4 and YY1.

After demonstrating that YY1 regulates the expression of KLF4, we explored the effects of inhibiting YY1 expression using siRNA to target YY1. Ramos lymphoma cells were transfected, and, as shown in Figure 2A, we demonstrated the inhibition of YY1 by western blotting with the lysates of cells transfected with siRNA-YY1, while the respective controls did not exhibit inhibition. As expected, the expression of KLF4 decreased proportionally with YY1 expression. We performed a functional experiment to corroborate that YY1 modulates KFL4 promoter through cells transfected with commercial KLF4-Luc reporter treated with siRNA YY1; as expected siRNA YY1 inhibited Luc activity of KLF4-Luc reporter (Figure 2B).

To determine the localization of YY1 and KLF4 expression, we performed immunofluorescence assays using specific antibodies for KLF4 and YY1. In Figure 2C, we show representative microphotography of immunofluorescence staining, which clearly shows a decrease in nuclear expression of YY1 and KLF4 after transfection with siRNA YY1.

### Confirmation of the interaction of YY1 with the KLF4 promoter through transcription factor profiling assays

**Figure 3:**
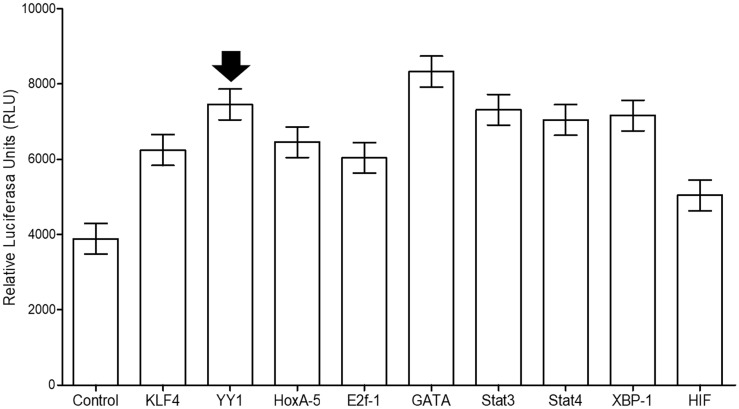
Transcription factor (TF) assay shows 9 of the 96 transcription factors found in the microarray plate The results indicate TFs exhibiting statistically significant differences (*p* > 0.001). The TFII transcription factor was used as a control, as suggested by the manufacturer (Signosis™).

As mentioned in the introduction, it has been reported that different transcription factors are associated with the transcriptional regulation of KLF4. In this study, using a commercial kit, we established ability of YY1 to regulate KLF4, and this ability correlated with that of other transcription factors that are capable of binding to the KLF4 promoter, in addition to those previously reported. The plate microarray contained 96 transcription factors that were tested for their ability to bind to the KLF4 promoter, which obtained from a commercial reporter plasmid (Switchgear, Genomics [S722399]). We employed a control to measure the relative expression of TFIID (suggested by the manufacturer). Figure 3 shows nine representative transcription factors that significantly bound to the KLF4 promoter in Ramos cells. These include STAT3 and HoxA5, which have been associated with cancer induction. STAT4 is required for the development of Th1 cells. GATA is an important transcription factor involved in cancer cell growth, and E2F1 plays a crucial role in controlling the cell cycle. XBP-1 regulates autophagy and lipid accumulation. Gfi-1 plays important roles in hematopoiesis and oncogenesis. These results corroborate the ability of KLF4 to self-regulate its promoter, as described in the literature, and demonstrate that YY1 binds to the KLF4 promoter [14].

### Correlation of the expression of KLF4 and YY1 in various B-NHL cell lines and tissues from patients with lymphoma

**Figure 4:**
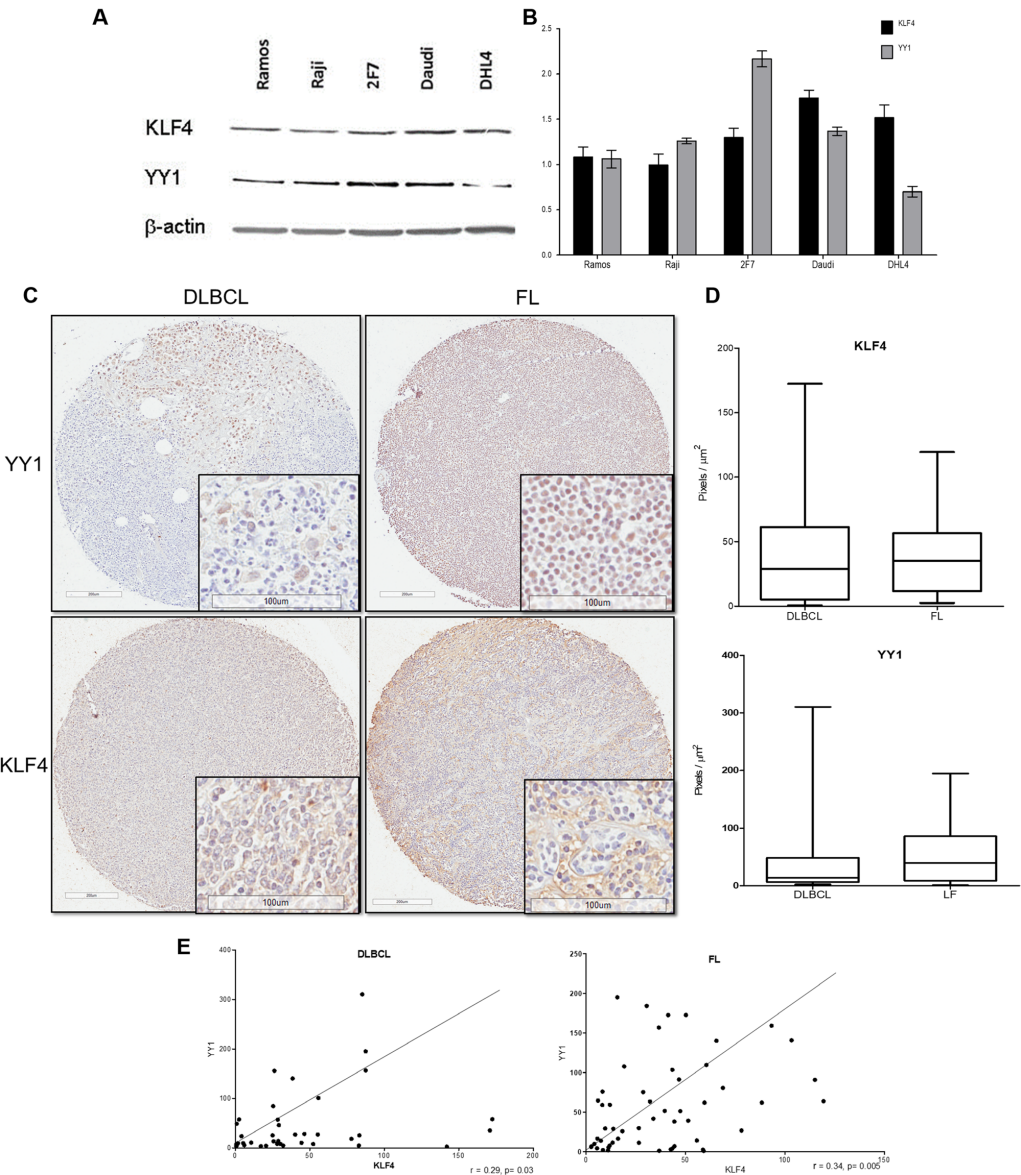
Expression of and correlation between KLF4 and YY1 in several tumor cell lines and NHL tissue arrays (**A**) Western blotting for KLF4 and YY1 expression was performed to analyze several B-NHL cell lines. The signals in each KLF4 and YY1 western blot were normalized to b-actin levels and then expressed in relative arbitrary units of expression over b-actin as a loading control; the bars represent the densitometry analysis (**B**). (**C**) Analysis of KLF4 expression in NHL TMAs by IHC. Follicular and DLBCL samples are shown. The right panel shows representative images of total staining of KLF4 and YY1 (original magnification: × 100 and; × 400 in the frame). (**D**) The IHC staining intensities of the anti-KLF4 and anti-YY1 antibodies were scored, and the relative intensities of positive cells were calculated using digital pathology. The data demonstrate that the follicular histological subtype shows statistically higher KLF4 expression than the DLBCL histological subtype (^*^p < 0.05). (**E**) Expression correlation was analyzed for KLF4 and YY1 in both subtypes using the relative intensity data. All tumor samples were analyzed for KLF4 and YY1 expression by IHC.

We examined the expression of KLF4 and YY1 in several B-NHL cell lines by western blot. The findings, shown in Figure 4A, demonstrate that expression of KLF4 correlated with YY1 expression in Ramos, Raji, and Daudi cells, and expression of YY1 is higher than KLF4 in 2F7, and an inverse pattern was observed in DHL4 which were shown to have high KLF4 and low YY1 expression. Definitively the expression of KLF4 and YY1 seem to correlate in most of the cell lines analyzed. In previous work reported by us and other authors KLF4 and YY1 independent expression does not depend on the subtype of lymphoma; however, patient samples showed a correlation as described by us and other authors. [20, 21] The relative expression of KLF4 and YY1 was analyzed by densitometry (Figure 4B). These findings demonstrate that KLF4 and YY1 expression was correlated in several tested B-NHL cell lines.

In addition, as previously mentioned, it has been shown that the expression of KLF4 and YY1 in lymphoma is correlated with low patient survival [20, 23]. In this study, we analyzed the expression of both proteins in biopsies from patients included in a TMA using immunohistochemistry and a digital pathology assay, and then we analyzed the correlation of that expression. The total numbers of biopsies positive for each analyzed protein (KLF4 and YY1) by immunohistochemistry are shown in Table 1. In total, 76% of patient samples were positive for KLF4 expression, while YY1 was expressed in 65% of patients.

**Table 1:** Expression of YY1 and KLF4 in samples from NHL patients

Protein	Frequency (%)
YY1	
Positive	28 (65)
Negative	15 (35)
KLF4	
Positive	33 (76)
Negative	10 (24)

We analyzed the expression of KLF4 and YY1 for each subtype of lymphoma included in the TMA (Table 2). Interestingly, we found that 69% of follicular lymphomas were positive for KLF4, and 65% were positive for YY1, while for DLBCL, KLF4 expression was found in 88% of samples and YY1 expression in 65% of samples. [24]. Similar to another report on lymphoma follicular, we found YY1 expression in a total of 65% of samples (17/26) [24].

**Table 2:** Expression of YY1 and KLF4 in subtypes of NHL biopsies

Subtype of lymphoma	Frequency (*n* = 43)	Percentage (%)	KLF4 + (*n* = 43)	KLF4 – (*n* = 43)	YY1 + (*n* = 43)	YY1 – (*n* = 43)
Follicular	26	60.5	18	8	17	9
lymphoma DLBCL	17	39.5	15	2	11	6

In Figure 4C, we show representative microphotographs of KL4 and YY1 staining in the TMA for DLBCL and FL. The expression of both proteins was primarily nuclear but was also present in the cytoplasm. Interestingly, expression in FL was higher than in DLBCL. We analyzed the total expression (nuclear and cytosol) of KLF4 and YY1 in the TMA using digital pathology, as described in the Materials and Methods. The results demonstrated that the expression of both YY1 and KLF4 was significantly higher in FL (^*^p < 0.005). (Figure 4D). We analyzed the correlation between the expression of both proteins for each subgroup (follicular and DLBCL), and we found a positive correlation in both subtypes. However, the correlation was more significant in the follicular subtype (*p* < 0.005, *r* = 0.34), as shown in Figure 4E. These findings clearly demonstrate that the expression of KLF4 and YY1 was significantly correlated in the lymphoma samples analyzed.

### Network analysis of YY1/KLF4 and the construction of gene networks related to function

To demonstrate the functional interactions between KLF4 and YY1 and other transcription factors, a bioinformatics analysis was performed with GeneMANIA using the free software Cytoscape 2.8, which visualizes biological networks and integrates data, and the database Oncomine. Typically, annotations used by Cytoscape correspond to the GEO database (Gene Ontology Database). Both databases permit free access to microarray banks and data meta-analyses and networks, which allow the prediction of interactions between genes or proteins of interest.

**Figure 5:**
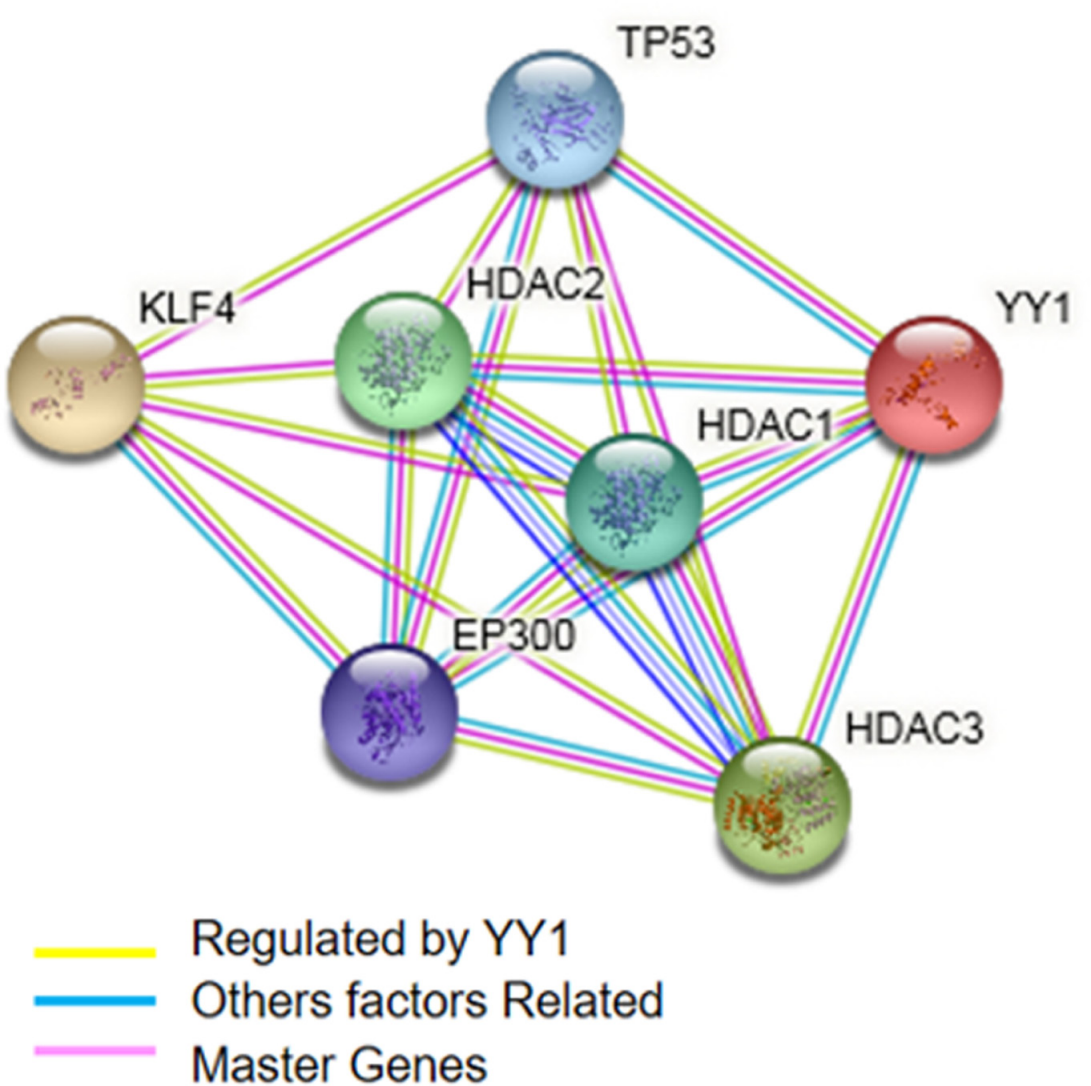
Prediction of the biological interaction between KLF4 and YY1 (highlighted in light yellow line) The figure indicates the inferred GeneMANIA network program regulated by the YY1 (bright yellow) gene network. Additionally, other factors related to transcription (blue) were inferred. The pink factors are “master genes” or hubs that regulate at least three genes.

According to a meta-analysis performed with the microarray databases, KLF4 and YY1 share zinc finger protein domains. However, we also identified co-activators such as HDAC1, 2 and 3, which belong to the histone deacetylase family. Histone deacetylases act via the formation of large multiprotein complexes and are responsible for the deacetylation of lysine residues at the N-terminal regions of core histones. These proteins form transcriptional repressor complexes by associating with many different proteins, including YY1 and probably KLF4, or by playing a role in their regulation (Figure 5). Additionally, the meta-analysis showed an interaction with TP53, a tumor suppressor protein containing transcriptional activation, DNA binding, and oligomerization domains. The encoded protein responds to diverse cellular stresses to regulate the expression of target genes, thereby inducing cell cycle arrest, apoptosis, senescence, DNA repair, or changes in metabolism, and can interact directly or indirectly with the YY1/KLF4 complex (Figure 5).

### Bioinformatics analysis of and correlation between KLF4 and YY1 gene expression in B-cell lymphomas

**Figure 6:**
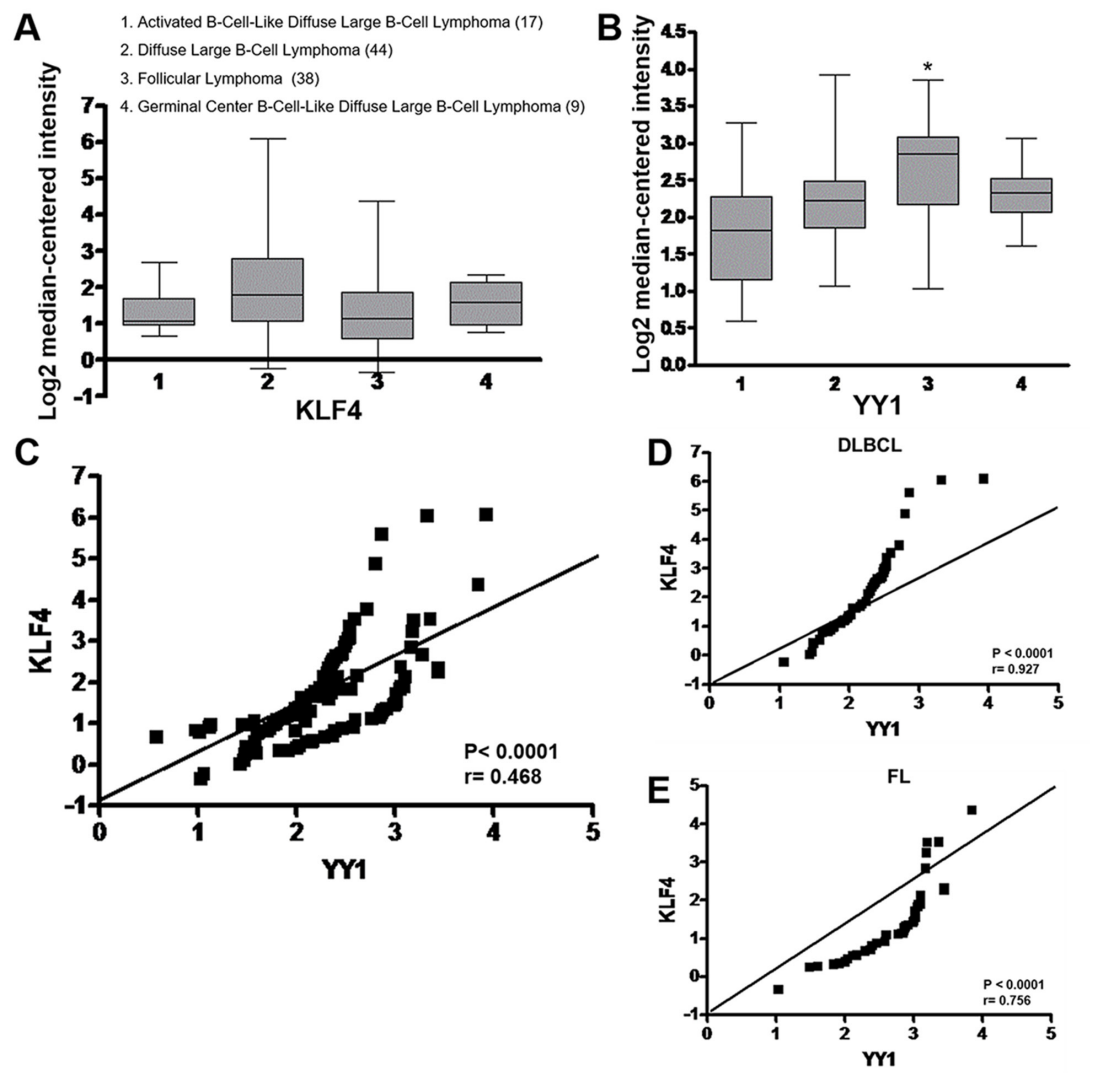
Gene expression and correlation of KLF4 and YY1 in B-NHL (**A**) Analysis of KLF4 and YY1 expression levels in several subtypes of B-NHL was performed using a public dataset of microarrays retrieved from the Oncomine database and the Gene Expression Omnibus NCBI gene expression and hybridization array data repository, obtained from an analysis from Compagno *et al*. [25]. The results are shown as boxed quartiles (median, 25th, and 75th percentile) and whiskers (minimum and maximum). (**A**, **B**) Oncomine™ boxed plot of KLF4 and YY1 expression levels among different types of NHL from the datasets reported in studies by Compagno *et al*. (^*^p < 0.05). (**C**) KLF4 gene expression levels in ABC-DLBCL, DLBCL, GC-DLBCL and FL cells correlated with YY1 expression (^*^p < 0.0001 *r* = 0.468). (**D, E**) KLF4 and YY1 expression correlation were analyzed for DLBCL and FL (*p* = 0.001 *r* = 0.927 and *r* = 0.756 respectively).

An analysis of KLF4 and YY1 expression levels in different subtypes of B-cell lymphomas was performed using a public data set of microarrays retrieved from the Oncomine and Gene Expression Omnibus databases, derived from a published analysis reported by Compagno *et al.* [25] The microarray data were from 108 tumor samples out of the 136 samples present in a related data set. These samples comprised 17 ABC-DLBCL, 44 DLBCL, 38 FL and 9 GC-DLBCL samples. All tumors showed the expression of KLF4 and YY1 (Figure 6A, 6B). and were analyzed selectively for KLF4 and YY1 co-expression, and a significant correlation was found for all DLBCL tissues as well for FL tissues. As shown in the Figure 6C, KLF4 gene expression in DLBCL and FL samples correlated with YY1 expression (^*^p < 0.0001, *r* = 0.468). In FL in particular, a greater expression of YY1 compared to KLF4 is observed, which is consistent with other studies [24]. However, FL shows a positive correlation similar to that observed in DLBCL (*r* = 0.756 *vs r* = 0.927 *p* < 0.001 respectively). (Figure 6D) [26].

## DISCUSSION

KLF4 is a transcription factor that plays a crucial role in cellular proliferation. Because different types of cancer show dysregulated cell growth, KLF4 has been considered a key factor in cancer development and progression. This transcription factor is capable of inducing cell growth arrest, and it can be assumed that KLF4 possesses anti-carcinogenic activity. KLF4 demonstrates low expression in a variety of cancers and therefore has been considered a tumor suppressor. However, its role in cancer has not been defined conclusively, as it also has been identified as an oncogene in some tissue-specific cancers; for example, KLF4 has been identified as an oncogene and linked increased expression in epithelial carcinomas of the oral cavity.

KLF4 can play the role of an oncogenic promotor via p53 inhibition to induce oncogenic transformation. In the absence of cyclin D1 and p21, KLF4 induces the elevated expression of p21, c-Myc and cyclin D2. KLF4 can regulate the cell cycle in B-cell malignancies [27]. KLF4 expression has been reported in different leukemia and B-lymphocyte lymphoma cell lines. Additionally, we have previously reported the expression of KLF4 in biopsies of pediatric patients and its correlation with poor prognosis. However, the possible regulatory mechanisms in different types of cancer have been poorly studied. In this work, we report for first time the transcriptional regulation of KLF4 by YY1, and we demonstrate a correlation between YY1 and KLF4 expression in biopsies from patients with NHL. Recent studies have demonstrated the constitutive expression of YY1 in lymphoma, and its expression correlates with some subtypes of more aggressive lymphomas. However, other studies have reported no correlation between YY1 transcription levels and patient survival. [28] The biological implications of YY1 and this correlation are unknown, so in this study, we analyzed the correlation between YY1 and KLF4 expression in biopsies of patients with lymphoma and evaluated the possible regulation of KLF4 expression by YY1. In an *in vitro* model using a cell line derived from Burkitt lymphoma (Ramos), we showed high expression of KLF4 and YY1, and the KLF4 promoter contained two consensus sites for YY1 binding. ChIP assays showed that these two sites, at -950 bp and -105 bp, were positive for YY1 binding, suggesting that YY1 transcriptionally regulates KLF4 expression (Figure 1), and inhibition of YY1 expression by interfering RNA resulted in the inhibition of KLF4 expression in this model (Figure 2).

Assays performed in cell lines showed that YY1 regulates KLF4 expression, and the inhibition of constitutive YY1 expression by siRNA also decreased KLF4 expression, suggesting that YY1 can regulate KLF4 expression. ChIP analysis showed that YY1 directly binds to the KLF4 promoter at both potential sites, as determined by computational analysis using TESS. There have been no previous reports showing that YY1 can regulate KLF4 by directly interacting with its promoter. Thus, to evaluate the effects of the transcriptional regulation of YY1 on the KLF4 promoter, we constructed a reporter plasmid assay (luciferase) in which we cloned the KLF4 promoter and subsequently performed site-directed mutagenesis to delete each YY1 binding site. The results showed that removing the YY1 sites affected reporter gene expression.

Our previously results have shown that YY1 inhibition by siRNA leads to the inhibition of KLF4 expression, which correlates with decreased cellular proliferation and apoptosis induction; these results have been corroborated by KLF4 chemical inhibition (with kenpaullon) (final manuscript in preparation) [29].

Bioinformatics analysis with Cytoscape permits the identification of active subsets/modules. A network is analyzed in conjunction with gene expression databases (microarray databases used in this study: ONCOMINE, GEO-NCBI) to identify sets of connecting interactions between proteins, that is, to call interaction subsets in which genes show particularly high levels of differential expression. The interactions contained within each subset provide hypotheses for regulatory and signaling interactions controlling observed changes in expression. One can search groups (highly interconnected regions) and load any network in Cytoscape. Depending on the type of network, groups can have different meanings. Networks are designed with automated algorithms. Our Cytoscape analysis identified interactions between KLF4 and YY1, and this correlation was confirmed by experimental findings obtained with ChIP and binding site mutation, but interestingly, Cytoscape analysis also revealed that KLF4 and YY1 are “HUBS” or nerve centers involved in regulating other transcription factors. Furthermore, KLF4 and YY1 may be involved in various central regulatory mechanisms associated with organismal physiology. Categorization by GeneMANIA software revealed genes that are transcriptionally regulated and relevant in the process of cell proliferation, such as TP53 and KLF4. [30] Conversely, YY1 is related to epigenetic regulation. Thus, it is important to study these molecular mechanisms because these patterns indicate a possible regulatory role for KLF4 either in normal physiological processes or certain pathologies.

The TF array assay demonstrated that other TFs can regulate KLF4, both in normal physiological processes or in pathological processes. Among these transcription factors was STAT3, which can have an oncogenic or tumor suppressor role depending on the mutational status of the tumor or the methylation status of the HoxA5 promoter, resulting in a loss of expression. As STAT3 regulates p53, this protein may have a role in the genesis of cancer. E2F1 regulates the cell cycle and is involved in cancer genesis [31], and the GFi-1 protein regulates hematopoiesis and oncogenesis. [32] The XBP1 protein, which is alternatively transcribed, can generate autophagy or control lipid accumulation through the PI3K/AKT pathway [31, 33].

We observed that transcription factors either regulate normal physiology or are dysregulated in some physiological and pathological processes; however, there are no proteins that bind to promoters and initiate transcription and act together with other transcription factors that, in turn, are co-transcriptionally processed during alternative splicing, post-transcriptionally by ubiquitination or sumoylation, or undergo epigenetic regulation. Additionally, such transcription factors act in concert with other factors. Recently, it has been shown that miR-29 expression in rhabdomyosarcoma is lost via inhibition due to NF-kB and YY1 “expression forming” a state of uncontrolled regulation. [34] KLF4 has been considered to be an oncogene only in breast cancer. However, there is opposing evidence. A meta-analysis based on the database ONCOMINE found that KLF4 transcript levels are lower in this type of cancer. Given the evidence that KLF4 may be involved in a central mechanism in cancerous cells that are activated depending on the environment and the cellular context, it is important to check the intratumoral heterogeneity of KLF4 and examine its apparently Darwinian adaptation during convergent evolution [35]. To corroborate our results *in vitro*, we evaluated YY1 and KLF4 expression in patients with lymphoma. Our results demonstrated the significant expression of both proteins in lymphoma patients. Furthermore, this expression was directly proportional. This result is interesting, considering that no previous studies have shown the involvement of YY1 in the pathophysiology of lymphoma. Our results clearly show the constitutive expression of KLF4, strongly suggesting the importance of KLF4 protein expression in the pathogenesis of lymphoma. However, it is clear that KLF4 activation via specific phosphorylation of SER-123 is needed for this protein to perform its biological activities in a wide range of cellular processes, including cell growth, differentiation, and apoptosis.

In this study, we determined KLF4 expression levels by IHC and analyzed this expression in malignant cells. The results indicated that a total of 69% of follicular lymphomas expressed KLF4 (Table 2 and Figure 5), and 88% of DLBCL lymphomas expressed KLF4.

Based on data retrieved from Oncomine, we found that YY1 and KLF4 mRNA were expressed in several lymphoma subtypes and in most high-grade lymphoma tumors, such as follicular or DLBCL lymphomas, as shown in Figure 6. Interestingly, we found a positive correlation between the expression of KLF4 and YY1 in several data sets analyzed from the lymphoma study by Campagno [25]. This correlation is consistent with the findings from our *in vivo* patient samples and confirms the interaction and regulation of KLF4 by YY1. These findings indicate that YY1 and KLF4 might participate in the initiation as well as the progression of lymphoma via transcriptional regulation.

This is the first report describing a correlation between KLF4 and YY1 expression in lymphoma, and this study identifies KLF4 and YY1 as potential disease markers, which could be considered biomarkers at the time of diagnosis for predicting disease behavior. We also propose that the use of pharmacological or chemical inhibitors targeting YY1 and KLF4 could be an alternative treatment for patients with lymphoma that are known to be positive for YY1 and KLF4 expression, thus offering a therapeutic alternative for this disease.

## MATERIALS AND METHODS

### Cell lines

Ramos, Raji, 2F7, Daudi, DHL4 NHL, and PC3 cell lines were purchased from the American Type Culture Collection (ATCC, Manassas, VA). Cells were grown in RPMI 1640 (Mediatech, Cellgro) supplemented with 10% heat-inactivated fetal bovine serum (Life Technologies, Invitrogen Co.) and 1% bacteriofungicide solution containing 10,000 U/mL penicillin G, 10 mg/mL streptomycin, and 25 μg/mL fungizone (Cellgro). Cells were cultured in 5% CO2 at 37° C. All cells used in this study were used within 15 passages after resuscitation. The cells were checked routinely for morphology and tested for mycoplasma contamination with the CELLshipper^®^ Mycoplasma detection Kit (Bionique® Testing Laboratories, Saranac Lake, NY).

### Transcription factor profile assays

For transcription factor (TF) assays, we used the commercial TF Activation Profiling Plate Array II Kit (Signosis™) following the recommendations of the manufacturer. Briefly, labeled probes were marked with biotin and hybridized with the consensus sequence for each TF´s DNA. The mix was incubated with isolated KLF4-promter region from commercial KLF4-Luc promoter (Switchgear Genomics (S722399). Sequenced reported at: https://switchdb.switchgeargenomics.com/productinfo/id_722399/); individual probes found each corresponding TF and formed complexes. TFs/probes were easily separated by a purification method or through separation columns. The probes were separated and analyzed by hybridization on plates, and every plate well was specifically covered with complementary sequences to the probes. The captured DNA probes were detected with streptavidin-HRP. Luminescence was reported as light relative units (URLs) in a microplate luminometer.

### Network analysis between YY1/KLF4 and the deduction of functional gene-related networks

To develop this analysis, the software Cytoscape 3.1 (https://cytoscape.org) [36] and the database Oncomine (https://www.oncomine.org) [37] were used to visualize biological networks and integrate data. The annotations used by Cytoscape correspond to the GEO database (Gene Ontology Database) (https://www.ncbi.nlm.nih.gov/geo) [36]. Both Oncomine and GEO are microarray database repositories that can be used to predict interactions between genes or proteins. The algorithm used by Cytoscape is the weighted sum of each network, which is determined based on its predicted function [38].

### Determination of YY1 binding sites in the KLF4 promoter by *in silico* analysis

YY1 binding sites in the KLF4 promotor were predicted with the programs TESS (Transcription Element Search System), PROMO and JASPAR. TESS combines the TRANSFAC database v6.0, JASPAR 20060301, IMD v1.1 and CBI/GibbsMat v1. PROMO uses the complete TRANSFAC collection database, and JASPAR is supported by its own database, JASPAR CORE. Two thousand nucleotides were analyzed upstream of the ATG codon (GeneBank No. DQ658241.1) gene promoter sequence and 151 nucleotides downstream. Two putative sites were located at −950 bp and −105 bp with respect to the transcription start site.

### Western blot

Cell lines were lysed, electrophoresed in 12% sodium dodecyl sulfate – polyacrylamide gel electrophoresis (SDS – PAGE) gels (Bio-Rad, Hercules, CA) and transferred to nitrocellulose membranes. Membranes were blocked in an Odyssey blocking buffer (Li-cor, Lincoln, NE, USA) for 1 hour. Membranes were incubated with the primary antibodies anti-KLF4 (B-9, SC1661001) (Santa Cruz Biotechnology, Inc. Santa Cruz, CA) and anti-YY1 (H-10, SC-7341) (Santa Cruz Biotechnology, Inc.). Levels of b-actin were used to normalize the protein expression. Membranes were incubated with a secondary antibody coupled to IRDye 680LT goat anti-rabbit fluorescent particles (Li-cor, Lincoln, NE, USA) in a blocking solution for one hour. Finally, the membranes were analyzed and documented with the CLx ODYSSEY infrared imaging system by Li-Cor (Li-cor, Co. Nebraska USA).

### Cloning of the promoter region of KLF4

Genomic DNA was isolated from peripherical blood mononuclear cells using TRIzol (Invitrogen, Thermo Fisher Sc. USA), as suggested by the manufacturer. This DNA was used as a template to amplify the promoter region of the KLF4 gene (−2000 bp to +400 bp relative to the start site for gene transcription) by PCR using the following specific primers: sense primer, 5′-ctgcaggagagtgcgtggcttgaaaagtcat-3′ (underlining indicates Pst1 site), and anti-sense primer 5′-ggatccacagctgag ccaaggacacggaag-3′ (underlining indicates BamH1). Once the promoter sequence of KLF4 was amplified, it was purified and cloned into the vector pJet, generating the construct pJet-promoter-KLF4. Subsequently, the promoter sequence of KLF4 was subcloned from the vector pJet into pGL3 (Promega, Co. USA) using the restriction enzymes Pst1/BamH1. Briefly, the pJet-promoter-KLF4 construct was digested with the restriction enzymes indicated above. After that, the fragment of interest was purified and ligated into the pGL3 vector, which was previously treated with the same restriction enzymes to generate the pGL3-promoter-KLF4 construct. The ligation product was transformed into E. coli DH5a. We verified the identity of the pGL3-promoter-KLF4 plasmid by obtaining endonuclease restriction maps.

### Site-directed mutagenesis of putative binding sites for YY1 in the KLF4 promoter

As mentioned above, we identified two putative binding sites for YY1 in the KLF4 promoter. We performed site-directed mutagenesis using the QuickChange Lighting Site-Directed Mutagenesis kit from Agilent Technologies. A computer algorithm provided by the manufacturer was used to design specific primers to introduce the desired mutations. Incorporation of the previously indicated mutations was performed by PCR using the primers described in the Table 3. The manufacturer's instructions were followed. Briefly, we performed a PCR reaction in which the template was the pGL3-promoter-KLF4 construct, using each set of primers mentioned in Table 3. The following PCR conditions were used: 94° C 4 min, 94° C 30 sec, 68° C 30 sec, and 72° C 1.20 min, for 35 cycles, finally 72° C 5 min. After enzymatic digestion with the restriction enzyme Pvull, we transformed the PCR-amplified products into E. coli DH5a. Bacteria containing mutations induced by mapping the restriction enzymes were selected.

**Table 3:** Sequence of primers used for site-directed mutagenesis

Primer	Sequence	Modification
KLF4 Prom Anti-sen	5′- CTGCAGAGAGTGCGTGGCTTGAAAAGTCAT-3′	
KLF4 Prom Sen	5′- GGATCCACAGCTGAGCCAAGGACACGGAAG-3′	
Sitio A WT	5′-TTCTTCGACCCGGGAGTGGGCCGAGATTGCAGCGCTGGCGCCCTG GGTTCCC-3′	
Mut A Sen	5′-GCGCGACCAGGGCCGTACTCACAAAAGCTTTCGGCTCCCTGGGTT CGAAGCC-3′	HindIII
Mut A Anti-sen	5′-GGCTTCGAACCCAGGGAGCCGAAAGCTTTTGTGAGTACGGCCCT GGTCGCGC-3′	HindIII
Sitio B WT	5′-CACAAAAAAAAAAAAAAGAAAGAAAAAAAAAGGAAAGGGCTTC GAGATGGCTGGTTGAAAACTGTCTCCGCGC-3′	
Mut B Sen	5′-CGAAGAGAAGAAACGAAGCCAAAACCCAAAACCCCGGGAATTCC GAGATCCTTCTTCTTTGGATTAAATATAACTTG-3	EcoRI
Mut B Anti-Sen	5′-CAAGTTATATTTAATCCAAAGAAGAAGGATCTCGGAATTCCCGGG GTTTTGGGTTTTGGCTTCGTTTCTTCTCTTCG-3′	EcoRI

### Transfection of cell lines

PC3 cells were co-transfected with Lipofectamine 2000 (Invitrogen), following the manufacturer's instructions, with the reporter plasmid containing the wild-type promoter sequence of the promoter KLF4 (pGL3-KLF4-pro-luc) or different constructs modifying the YY1 mutant sites in the KLF4 promoter and pCMV-Sport-β-galactosidase vector (ThermoFisher Scientific), which is commonly used for determining transfection efficiency. Cells were co-transfected with 2 mg of total DNA at a 1:6 ratio (pCMV-Sport-β-galactosidase vector:pGL3-KLF4-pro-luc). At 48 hrs post-transfection, we extracted intracellular proteins to determine the enzymatic activities of the reporters involved. Luciferase and β-galactosidase enzymatic activity were determined using available kits (Promega and Clontech for luciferase and β-galactosidase activities, respectively). Quantification of luciferase and β-galactosidase activities was performed with a multimodal reader plate (EnSpire, Perkin Elmer).

### Chromatin immunoprecipitation (ChIP)

Chromatin immunoprecipitation was performed using a commercial kit (Active Motif) according the manufacturer's recommendations. Briefly, chromatin was obtained from 3 × 10^6^ Ramos cells and were fixed with 0.5% formaldehyde for 5 minutes. After that, 0.125 M glycine was added for 3 minutes to neutralize the reaction. The cells were then lysed in cell lysis buffer (10 mM EDTA, 50 mM TRIS-HCl, pH 8, 1% SDS, protease inhibitor cocktail). The cell lysate was sonicated to obtain soluble chromatin ranging between 200 and 400 bp. The pre-cleared lysate was subjected to chromatin immunoprecipitation (ChIP) using a specific antibody raised against YY1 (AB12132; Abcam). The DNA recovered after the ChIP assay was used as a template for PCR reactions with the following set of primers: detection at Site A, sense primer 5′-atgagtcacgcggataatcgcgc-3 and antisense primer 5′-tcgctgcgcgaccaggg-3′; detection at site B, sense primer 5′-aaagaagaaggatctcggcca-3′ and antisense primer 5′-gcgcctcacctacctcatta-3. And the following conditions: 94° C 4 minutes, 94° C 30 seconds, 68° C 30 seconds, then 72° C 1.20 minutes, repeat 35 times, finally 72° C 5 minutes and hold in 4° C. The PCR products (200 Kb) were analyzed on an agarose gel. The agarose gel was documented in a photodocumenter with a UV light transilluminator (Bio-imaging Systems, MiniBio Pro 2.0) and visualized using ethidium bromide. IP-blot was done to corroborate the efficiency of YY1 protein IP.

### Transfection with siRNA

Ramos cells were transfected with siRNA for YY1 and corresponding irrelevant controls by liposomes. Cells were treated with 30 nM siRNA YY1 (YY1 siRNA (h) SC-36863) or control siRNA (SC-36869) (Santa Cruz Biotechnology, Inc.) and transfected using Lipofectamine 2000 (Invitrogen, Thermo Fisher Scientific, USA) following the manufacturer's instructions. The transfected cells were incubated at 37° C and 5% CO2 for 48 hrs. Subsequently, a western blot was performed to determine the expression of YY1 and KLF4 in cells treated with siRNA.

### Immunohistochemistry and digital pathology in the TMA

Biopsies from 43 patients with NHL diagnosed in the Pathology Department of the Oncology Hospital UMAE “Siglo XXI” IMSS were included in this study. The pathology review was done by Dr. Isabel Alvarado, Dr. Ivonne Cuadra and M. A. Duran-Padilla using available records, which included institutional pathology reports and H&E stained slides. The samples were recruited collected by Dr. Natividad Neri and Dr. M. J Nambo and included 26 follicular lymphoma and 17 DLBCL subtypes. TMAs were constructed as previously reported [39]. Immunohistochemistry (IHC) was performed using a KLF4 or YY1 polyclonal antibody as previously reported [20].

Immunohistochemically stained sections were digitized at 40× magnification using an Aperio ScanScope CS (Aperio, Vista, CA). The Aperio ScanScope CS obtains 40× images with a spatial resolution of 0.45 μm/pixels. The images were reviewed using an ImageScope (Aperio). Once areas of interest were annotated, they were sent for automated image analysis using Spectrum Software (Aperio). For tissue intensity, an algorithm was developed to quantify total YY1 or KLF4 expression. The output from the algorithm returns a number of quantitative measurements, namely, the intensity, concentration and percentage of positive staining. Quantitative scales of intensity and percentage were categorized into 4 and 5 classes, respectively, after cut-off values were determined. The intensity of staining was categorized as 0 (no staining), 2+ (moderate) or 3+ (strong). The final IHC score was calculated from a combination of the intensity and percentage scores [40].

### Immunofluorescence

Rabbit anti-YY1 (Cell Signaling, Tech), Rabbit anti-KLF4 (Novus Biologicals), anti-rabbit IgG, Isotype control, AlexaFluor 488 Streptavidin (Jackson-lmmunoresearch, West Grove, CA, USA) and Vectashield-DAPI (Vector-laboratories, Burlingame, CA, USA) were used to stain Ramos cell lines transfected with siRNA-YY1. Images were acquired using a Leica TCS SP8x Confocal Microscope (Wetzlar, Germany) and were analyzed with Leica software.

### Sample analyses

A database of KLF4 and YY1 expression was created, and the information was processed using the statistics program Prisma © by GraphPad Software Inc. (San Diego, CA). The data were presented as arithmetic means with standard deviations. Evaluation of the differences in the numbers of positive cells and the density of the expression of IHC reactions was determined by one-way ANOVA. Multiple comparison analyses using Tukey's test were performed to identify the differences between groups. *p* ≤ 0.05 was considered significant. Monitoring was conducted by reviewing clinical records to document the response to chemotherapy.

### Comparative meta-profiling of mRNA expression data

The Oncomine Premium database (Oncomine Compendia Bioscience, Ann Arbor, MI) was used for analysis and visualization of bioinformatics analyses (https://www.oncomine.com) [24]. The differential expression of KLF4 and YY1 in existing NHL microarray datasets was analyzed by setting gene rank threshold values at 10% and *p* < 0.05.

### Statistical analysis

A database was developed, and information was processed using a statistical analysis program (Graph Pad Prism 4^®^ Software, Inc., San Diego, CA). Differences in the numbers of positive cells exhibiting immunocytochemical reactions were evaluated by performing analysis of variance (ANOVA). Correlation analysis was performed by using linear regression, giving an R-squared value with C.I. 95%.

## CONCLUSIONS

In conclusion, our findings demonstrate for the first time that YY1 regulates transcriptional KLF4, and inhibiting YY1 expression directly affects KLF4 expression in lymphoma. Furthermore, YY1 expression and KLF4 are increased in patients with lymphoma, and high expression of YY1 correlates with KLF4. Therefore, both KLF4 and YY1 may be possible therapeutic biomarkers in NHL.

## Abbreviations

B-NHL: B non-Hodgkin lymphoma
DLBCL: diffuse large B cell lymphoma
IHC: immunohistochemistry
KLF: Krüppel-like Factor
YY1: Ying Yang 1
